# ZKSCAN3 promotes bladder cancer cell proliferation, migration, and invasion

**DOI:** 10.18632/oncotarget.10679

**Published:** 2016-07-18

**Authors:** Takashi Kawahara, Satoshi Inoue, Hiroki Ide, Eiji Kashiwagi, Shinji Ohtake, Taichi Mizushima, Peng Li, Yi Li, Yichun Zheng, Hiroji Uemura, George J. Netto, Hitoshi Ishiguro, Hiroshi Miyamoto

**Affiliations:** ^1^ Departments of Pathology and Urology, Johns Hopkins University School of Medicine, Baltimore, MD, USA; ^2^ Department of Pathology and Laboratory Medicine, University of Rochester Medical Center, Rochester, NY, USA; ^3^ Departments of Urology and Renal Transplantation, Yokohama City University Medical Center, Yokohama, Japan

**Keywords:** bladder cancer, immunohistochemistry, tumor progression, ZKSCAN3

## Abstract

The expression status of ZKSCAN3, a zinc-finger transcription factor containing KRAB and SCAN domains, as well as its biological significance, in human bladder cancer remains largely unknown. In the current study, we aimed to determine the functional role of ZKSCAN3 in bladder cancer progression. Immunohistochemistry in tissue specimens detected ZKSCAN3 signals in 138 (93.2%) of 148 urothelial neoplasms, which was significantly higher than in non-neoplastic urothelial tissues [76 (84.4%) of 90; *P*=0.044]. Correspondingly, the levels of *ZKSCAN3* gene were significantly elevated in bladder tumors, compared with those in adjacent normal-appearing bladder mucosae (*P*=0.008). In a validation set of tissue microarray, significantly higher ZKSCAN3 expression was observed in high-grade and/or muscle-invasive urothelial carcinomas than in low-grade and/or non-muscle-invasive tumors. Two bladder cancer cell lines, UMUC3 and 647V, were found to strongly express ZKSCAN3 protein/mRNA, whereas its expression in 5637 bladder cancer and SVHUC normal urothelium cell lines was very weak. ZKSCAN3 silencing via its short hairpin RNA (shRNA) in UMUC3 and 647V resulted in significant decreases in cell viability/colony formation, cell migration/invasion, and the expression of matrix metalloproteinase (MMP)-2/MMP-9 and oncogenes c-myc/FGFR3, as well as significant increases in apoptosis and the expression of tumor suppressor genes p53/PTEN. ZKSCAN3 overexpression in 5637 also induced cell growth and migration. In addition, ZKSCAN3-shRNA expression considerably retarded tumor formation as well as its subsequent growth in xenograft-bearing mice. These results suggest that ZKSCAN3 plays an important role in bladder cancer outgrowth. Thus, ZKSCAN3 inhibition has the potential of being a therapeutic approach for bladder cancer.

## INTRODUCTION

Urinary bladder cancer remains one of the most commonly diagnosed malignancies worldwide, especially in elderly men [1]. More than two-thirds of newly diagnosed bladder tumors are superficial/non-muscle-invasive, which can typically be managed in a conservative manner. However, these patients with superficial disease are at high risk of recurrence, with occasional stage progression, after transurethral resection of the tumor and currently available prophylactic intravesical therapy. Additionally, despite available aggressive treatment options including radical cystectomy and systemic chemotherapy in neoadjuvant and adjuvant settings as well as their advances, the prognosis for muscle-invasive bladder cancer remains largely unimproved. Therefore, identification of key molecules responsible for the development and/or progression of bladder cancer is urgently required, which may successively provide novel targeted therapy [2].

ZKSCAN3 (ZNF306) is a family member of the KRAB and SCAN domain-containing zinc-finger transcription factors. It has been documented that this family of proteins is involved in important aspects of cellular functions, such as cell differentiation, cell proliferation, and apoptosis, as well as in cancer outgrowth [3, 4]. Indeed, ZKSCAN3 has been implicated in promoting the progression of a few types of malignancies, including colon cancer [5], multiple myeloma [6], and prostate cancer [7]. These malignancies have also been found to overexpress ZKSCAN3 [5–7]. Gene expression profiling has further suggested that downstream targets of ZKSCAN3 include cyclin D1 (CCND1), epidermal growth factor receptor (EGFR), insulin-like growth factor-2 (IGF-2), integrin-β4, NF-κB, and vascular endothelial growth factor (VEGF) [7, 8]. More recently, ZKSCAN3 has been shown to play a critical role in transcriptional regulation of autophagy [9, 10].

Frequent amplification of chromosome 6p22 on which the *ZKSCAN3* gene is located has been detected in various malignancies, including bladder cancer [11, 12]. In some of these studies assessing bladder cancer, strong associations between 6p22 gain and disease progression have been identified. However, the status of ZKSCAN3 expression in bladder cancer and its biological function in tumor progression remain largely unknown. In the current study, we investigated whether ZKSCAN3 was expressed in human bladder cancer and could affect its outgrowth.

## RESULTS

### Expression of ZKSCAN3 in human bladder cancer

**Figure 1:**
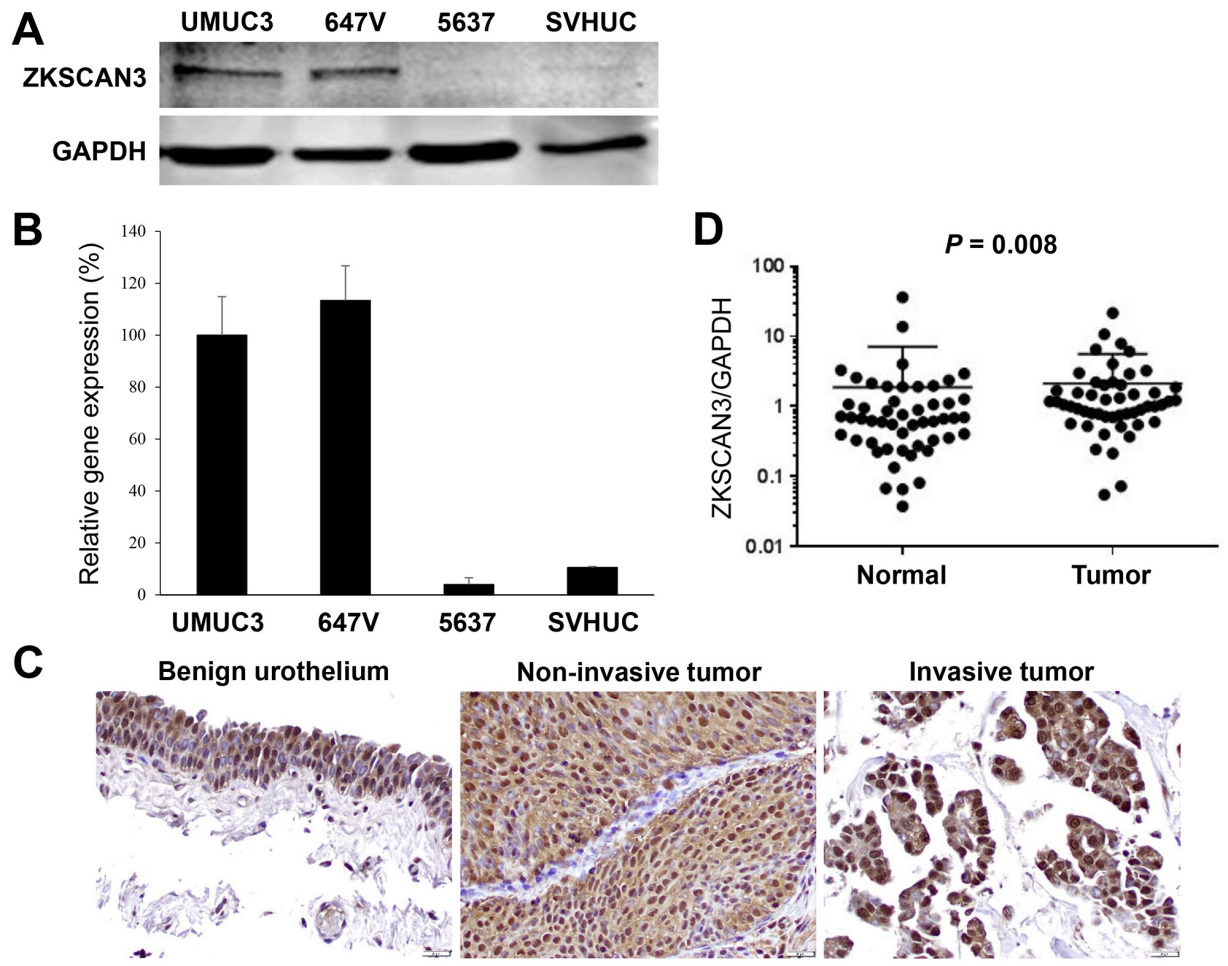
ZKSCAN3 expression in bladder cancer **A.** Western blotting of ZKSCAN3 in urothelial cell lines. Total protein extracted from each cell line was immunoblotted for ZKSCAN3 (60 kDa). GAPDH (37 kDa) served as an internal control. **B.** Quantitative RT-PCR of *ZKSCAN3* in urothelial cell lines. Total RNA isolated from each cell line was subjected to real-time RT-PCR. Expression of *ZKSCAN3* gene was normalized to that of *GAPDH*. Transcription amount is presented relative to that in UMUC3. Each value represents the mean (+SD) from at least three independent experiments. **C.** IHC of ZKSCAN3 in non-neoplastic urothelium, non-invasive urothelial tumor, and invasive urothelial tumor specimens. Original magnification, ×400. **D.** Quantitative RT-PCR of *ZKSCAN3* in non-neoplastic urothelium and urothelial tumor specimens. Expression of *ZKSCAN3* gene was normalized to that of *GAPDH*.

We first examined the expression of ZKSCAN3 in human bladder cancer cell lines, UMUC3, 647V, and 5637, as well as an immortalized human normal urothelial cell line, SVHUC, by western blotting (Figure 1A) and reverse transcription (RT)-polymerase chain reaction (PCR) (Figure 1B). ZKSCAN3 protein/mRNA expression was found to be strong in UMUC3 and 647V and very weak in 5637 and SVHUC.

We next stained immunohistochemically for ZKSCAN3 in a bladder tissue microarray (TMA) consisting of 148 urothelial neoplasm specimens and corresponding 90 non-neoplastic bladder tissues. Positive signals of ZKSCAN3 were detected predominantly in the nucleus of benign/malignant urothelial cells (Figure 1C). Overall, ZKSCAN3 was positive in 93.2% (29.7% 1+, 37.2% 2+, and 26.4% 3+) of tumors, which was significantly higher than in benign urothelial tissues [84.4% (30.0% 1+, 40.0% 2+, and 14.4% 3+)] (Table 1). However, there were no statistically significant associations between the status of ZKSCAN3 expression in bladder tumors and patient gender (male *vs.* female tumors), tumor grade [papillary urothelial neoplasm of low malignant potential (PUMLMP) + low-grade urothelial carcinoma *vs.* high-grade urothelial carcinoma], pT stage (non-muscle-invasive *vs.* muscle-invasive), or lymph node metastasis (pN0 *vs.* pN+). Kaplan-Meier and log-rank tests revealed no strong associations between ZKSCAN3 expression and a risk for the recurrence of non-muscle-invasive tumors (*P*=0.880), disease progression in all cases (*P*=0.170), or cancer-specific mortality in patients with muscle-invasive tumor who underwent radical cystectomy (*P*=0.678). For validating ZKSCAN3 expression in different grades and stages of bladder tumors, we stained another bladder cancer TMA (Supplementary Table S1). In this set of tissue specimens, strong (3+) ZKSCAN3 expression was detected in 12 (19.7%) of 61 low-grade carcinomas *vs.* 45 (45.9%) of 98 high-grade carcinomas (*P*=0.001) as well as in 18 (26.9%) of 67 non-muscle-invasive tumors *vs.* 39 (42.4%) of 92 muscle-invasive tumors (*P*=0.047).

**Table 1:** Correlation of ZKSCAN3 expression in bladder tumors with clinicopathologic profile of the patients

	n	Expression levels				*P*-value	
		Negative	Positive				
		0	1+	2+	3+	0 *vs* 1+/2+/3+	0/1+/2+ *vs* 3+
**Tissue**							
Non-neoplastic urothelium	90	14 (15.6%)	27 (30.0%)	36 (40.0%)	13 (14.4%)	0.044	0.036
Urothelial neoplasm	148	10 (6.8%)	44 (29.7%)	55 (37.2%)	39 (26.4%)		
**Gender**							
Male	113	5 (4.4%)	36 (31.9%)	42 (37.2%)	30 (26.5%)	0.057	1.000
Female	35	5 (14.3%)	8 (22.9%)	13 (37.1%)	9 (25.7%)		
**Tumor grade**							
PUNLMP + LG	53	2 (3.8%)	14 (26.4%)	18 (34.0%)	19 (35.8%)	0.333	0.055
HG	95	8 (8.4%)	30 (31.6%)	37 (38.9%)	20 (21.1%)		
**Tumor invasiveness**							
NMI	84	6 (7.1%)	21 (25.0%)	31 (36.9%)	26 (31.0%)	1.000	0.188
MI	64	4 (6.3%)	23 (35.9%)	24 (37.5%)	13 (20.3%)		
**Lymph node involvement**							
pN0	47	3 (6.4%)	17 (36.2%)	19 (40.4%)	8 (17.0%)	1.000	0.071
pN+	21	1 (4.8%)	6 (28.6%)	6 (28.6%)	8 (38.1%)		

PUNLMP: Papillary urothelial neoplasm of low malignant potential; LG: Low-grade urothelial carcinoma; HG: High-grade urothelial carcinoma; NMI: Non-muscle-invasive tumor; MI: Muscle-invasive tumor.

We then performed real-time RT-PCR to determine the expression of *ZKSCAN3* gene in 51 pairs of normal and neoplastic urothelial tissues. Overall, *ZKSCAN3* expression was significantly elevated in bladder cancer, compared with adjacent benign tissue (*P*=0.008; Figure 1D). High levels of *ZKSCAN3* expression in tumors than in normal tissues were seen in 38 (74.5%) of 51 cases. However, no statistically significant associations between the levels of *ZKSCAN3* expression and clinicopathologic features available for our patient cohort.

**Figure 2:**
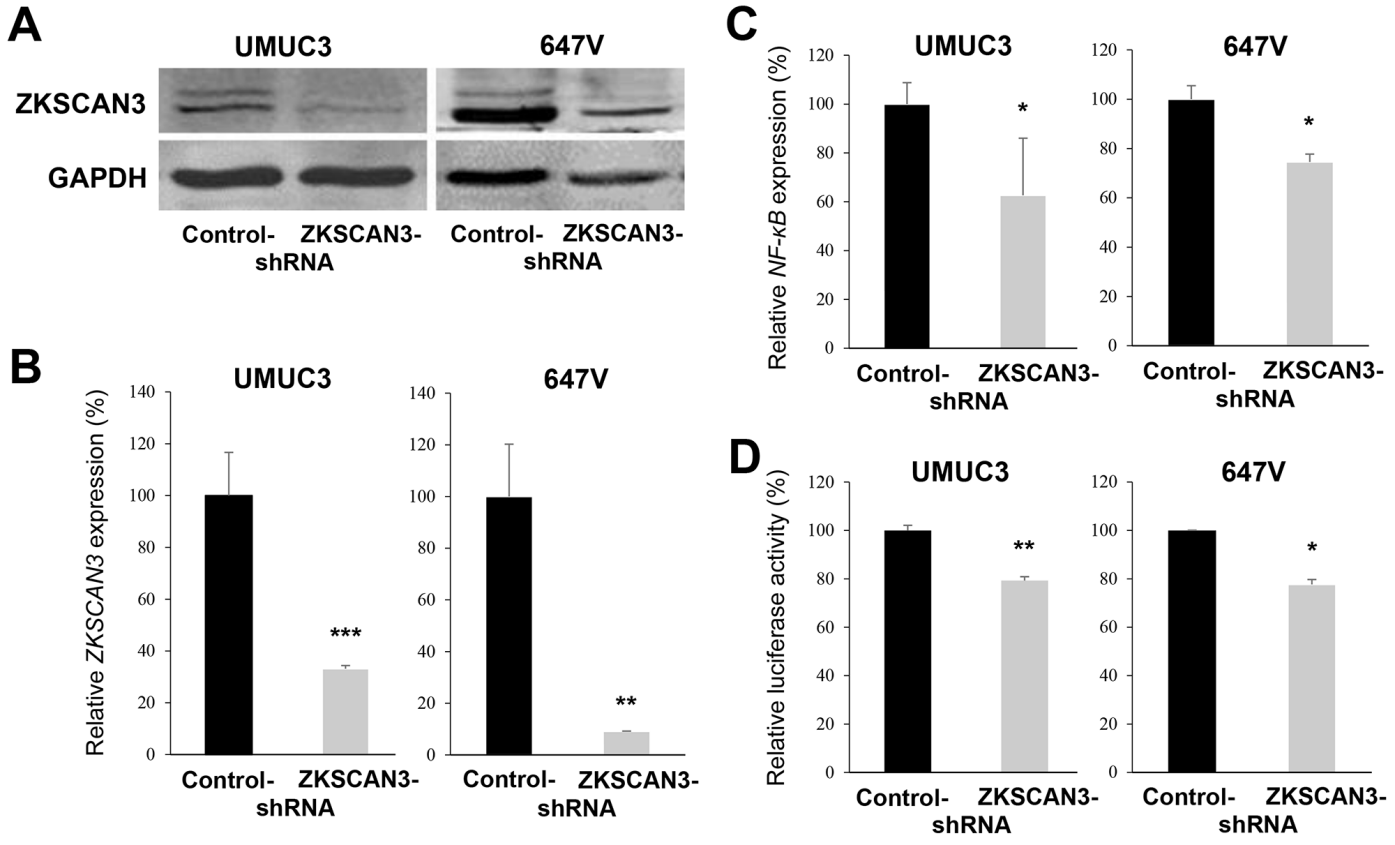
Inactivation of ZKSCAN3 in bladder cancer cells **A.** Western blotting of ZKSCAN3 in UMUC3-control-shRNA/ZKSCAN3-shRNA and 647V-control-shRNA/ZKSCAN3-shRNA. Cell extracts were immunoblotted for ZKSCAN3 (60 kDa). GAPDH (37 kDa) served as an internal control. Quantitative RT-PCR of *ZKSCAN3* **(B)** or *NF-κB* **(C)** in UMUC3-control-shRNA/ZKSCAN3-shRNA and 647V-control-shRNA/ZKSCAN3-shRNA. Expression of each specific gene was normalized to that of *GAPDH*. Transcription amount is presented relative to that of each control line. Each value represents the mean (+SD) from at least three independent experiments. **D.** NF-κB luciferase reporter activity in UMUC3-control-shRNA/ZKSCAN3-shRNA or 647V-control-shRNA/ZKSCAN3-shRNA transfected with NF-κB-Luc and pRL-TK. Luciferase activity is presented relative to that of each control line. Each value represents the mean (+SD) from at least three independent experiments. *P<0.05 (*vs.* control-shRNA). **P<0.01 (*vs.* control-shRNA). ***P<0.001 (*vs.* control-shRNA).

To further investigate the functional role of ZKSCAN3 in the growth of bladder cancer, a ZKSCAN3-short hairpin RNA (shRNA) was stably expressed in UMUC3 and 647V cells. As expected, the levels of ZKSCAN3 protein (Figure 2A) and mRNA (Figure 2B) were substantially lower in ZKSCAN3-shRNA-expressing lines than in respective scrambled control-shRNA-expressing lines. To confirm the down-regulation of ZKSCAN3 activity by its shRNA, we compared the expression and transcriptional activity of NF-κB, a known downstream target of ZKSCAN3 signals [7, 8], by a quantitative RT-PCR and a luciferase assay using a NF-κB reporter plasmid, respectively. The levels of NF-κB expression (Figure 2C) and its transcription (Figure 2D) were considerably diminished in ZKSCAN3-shRNA cells, compared with control-shRNA cells.

### Role of ZKSCAN3 in bladder cancer cell proliferation, migration, and invasion

**Figure 3:**
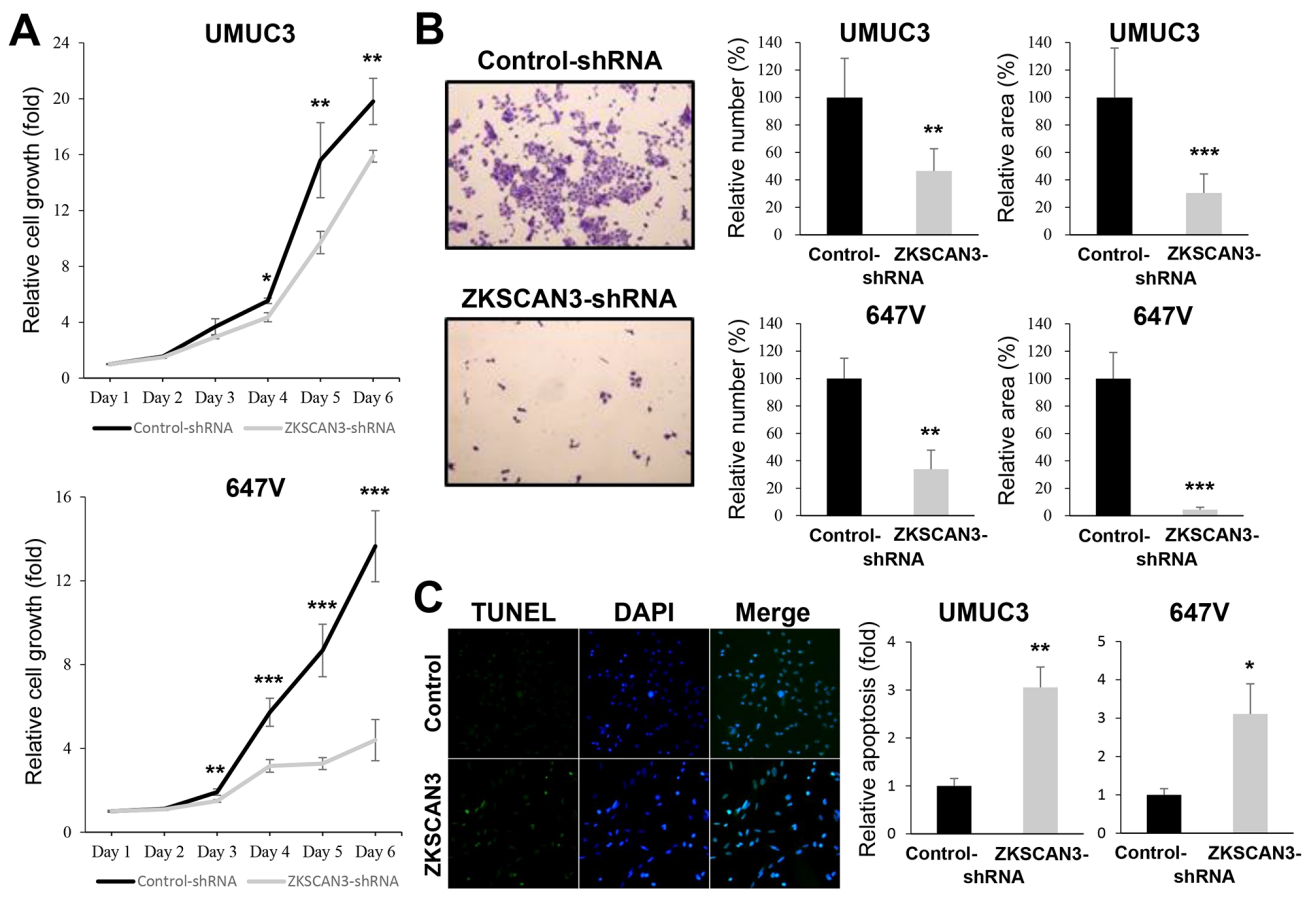
Effects of ZKSCAN3 inactivation on bladder cancer cell proliferation **A.** MTT assay in UMUC3-control-shRNA/ZKSCAN3-shRNA and 647V-control-shRNA/ZKSCAN3-shRNA cultured for 1-6 days. Cell viability is presented relative to that of each control line at day 1. Each value represents the mean (±SD) from at least three independent experiments. **B.** Clonogenic assay in UMUC3-control-shRNA/ZKSCAN3-shRNA and 647V-control-shRNA/ZKSCAN3-shRNA. The number of colonies and their areas quantitated, using the ImageJ software, are presented relative to those of each control line. Each value represents the mean (+SD) from at least three independent experiments. **C.** TUNEL assay in UMUC3-control-shRNA/ZKSCAN3-shRNA and 647V-control-shRNA/ZKSCAN3-shRNA. Apoptosis is presented relative to that of each control line. Each value represents the mean (+SD) from at least three independent experiments. *P<0.05 (*vs.* control-shRNA). **P<0.01 (*vs.* control-shRNA). ***P<0.001 (*vs.* control-shRNA).

To determine whether ZKSCAN3 down-regulation exerts an influence on the proliferation of bladder cancer cells, we compared cell viability [by methyl thiazolyl disphenyl tetrazolium bromide (MTT) assay] and colony formation (by clonogenic assay) between ZKSCAN3-positive lines versus their knockdown lines. The expression of ZKSCAN3-shRNA strongly suppressed the growth of UMUC3 (up to 31% decrease) and 647V (up to 68% decrease) cells at days 4-6 (Figure 3A). Similarly, ZKSCAN3 silencing resulted in significant decreases in the number and area of colonies in these cells (Figure 3B).

To investigate how ZKSCAN3 regulates cell proliferation, we performed terminal deoxynucleotidyl transferase-mediated dUTP nick end labeling (TUNEL) assay (Figure 5C) and flow cytometry (figure not shown) in ZKSCAN3-shRNA-expressing lines versus control-shRNA-expressing lines. In both assays, ZKSCAN3 knockdown was found to significantly induce apoptosis of UMUC3 (3.1-3.7–fold) and 647V (3.1-3.3–fold). However, it only marginally changed the cell cycle (*e.g.* G1 population) (data not shown).

**Figure 4:**
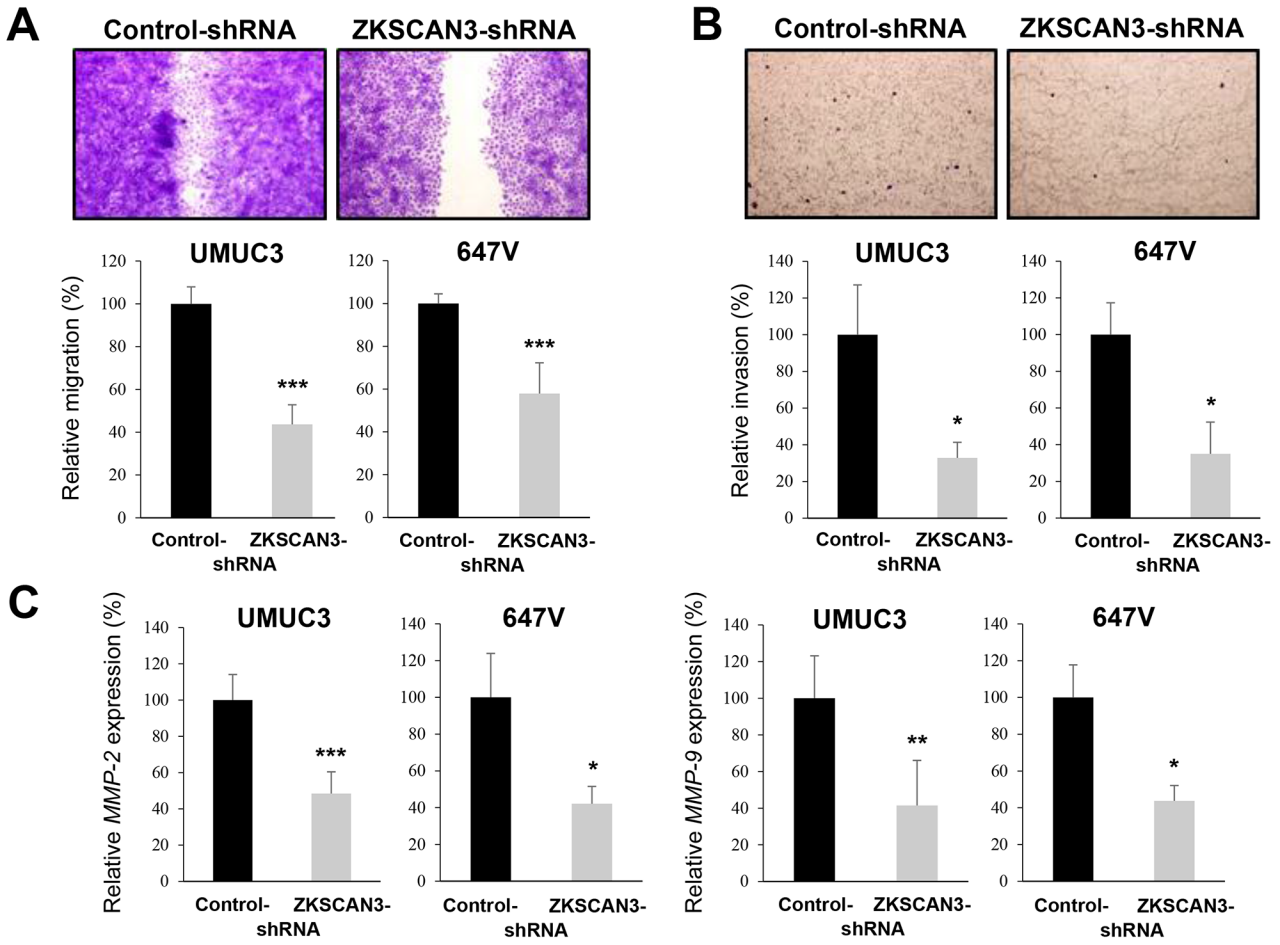
Effects of ZKSCAN3 inactivation on bladder cancer cell migration and invasion **A.** Wound healing assay in UMUC3-control-shRNA/ZKSCAN3-shRNA and 647V-control-shRNA/ZKSCAN3-shRNA. The cells grown to confluence were gently scratched and the wound area was measured after 24-hour culture. The migration determined by the rate of cells filling the wound area is presented relative to that of each control line. Each value represents the mean (+SD) from at least three independent experiments. **B.** Transwell invasion assay in UMUC3-control-shRNA/ZKSCAN3-shRNA and 647V-control-shRNA/ZKSCAN3-shRNA cultured in the Matrigel-coated transwell chamber. The number of invaded cells present in the lower chamber was counted under a light microscope (100× objective in five random fields). Cell invasion is presented relative to that of each control line. Each value represents the mean (+SD) from three independent experiments. **C.** Quantitative RT-PCR of *MMP-2* and *MMP-9* in UMUC3-control-shRNA/ZKSCAN3-shRNA and 647V-control-shRNA/ZKSCAN3-shRNA. Expression of each specific gene was normalized to that of *GAPDH*. Transcription amount is presented relative to that of each control line. Each value represents the mean (+SD) from at least three independent experiments. *P<0.05 (*vs.* control-shRNA). **P<0.01 (*vs.* control-shRNA). ***P<0.001 (*vs.* control-shRNA).

Cell migration and invasion are critical steps during tumor progression and metastasis. To see if ZKSCAN3 is involved in bladder cancer cell migration and invasion, we performed a scratch wound healing assay and a transwell invasion assay, respectively, in UMUC3 and 647V expressing either ZKSCAN3-shRNA or control-shRNA. In the wound healing assay, silencing of ZKSCAN3, compared with control cells, significantly delayed wound closure 24 hours after wound generation (Figure 4A). Similarly, in the transwell assay, knockdown of ZKSCAN3 demonstrated marked decreases in cell invasion ability, compared with control lines (Figure 4B).

Using a quantitative RT-PCR method, we then assessed the effects of ZKSCAN3 silencing on the expression of matrix metalloproteinases (MMPs) that are known to play a critical role in cancer cell migration/invasion, angiogenesis, and resultant tumor progression and metastasis. ZKSCAN3-shRNA significantly reduced the expression levels of *MMP-2* (Figure 4C) and *MMP-9* (Figure 4C), compared with control-shRNA, in two cell lines.

**Figure 5:**
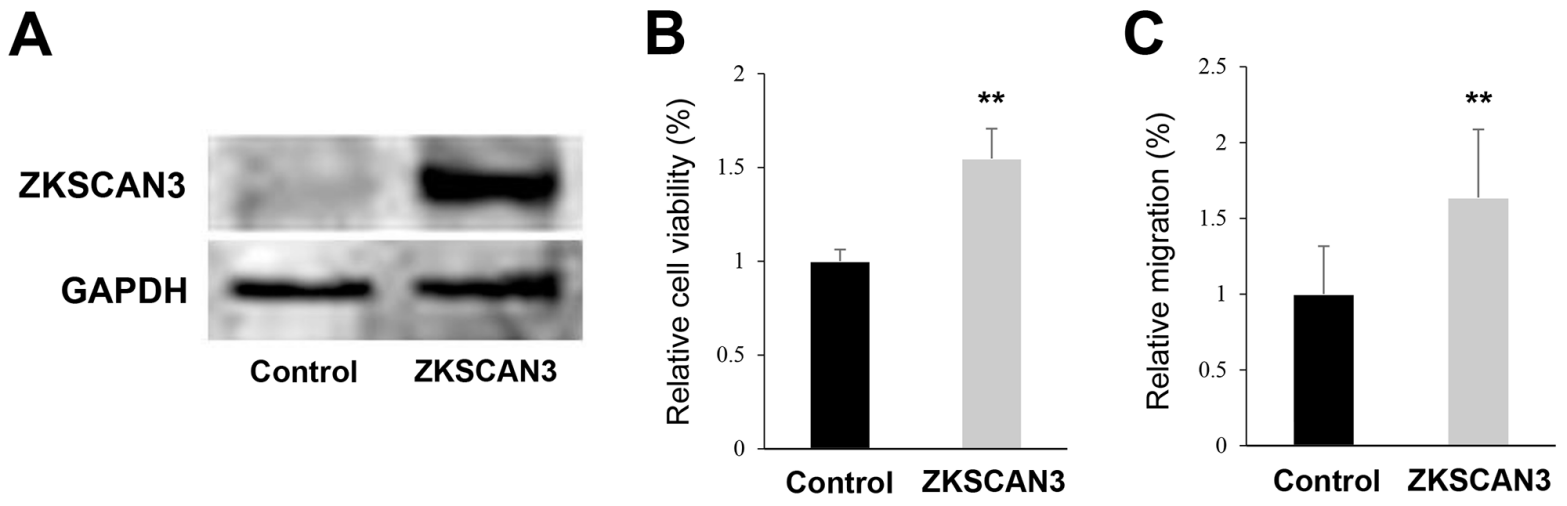
Effects of ZKSCAN3 overexpression on bladder cancer cell proliferation and migration **A.** Western blotting of ZKSCAN3 in 5637-control and 5637-ZKSCAN3 cells. Cell extracts were immunoblotted for ZKSCAN3 (60 kDa). GAPDH (37 kDa) served as an internal control. **B.** MTT assay in 5637-control and 5637-ZKSCAN3 cells cultured for 6 days. The viability of 5637-ZKSCAN3 cells is presented relative to that of 5637-control cells. Each value represents the mean (+SD) from at least three independent experiments. **C.** Wound healing assay in 5637-control and 5637-ZKSCAN3 cells. The cells grown to confluence were gently scratched and the wound area was measured after 24-hour culture. The migration of 5637-ZKSCAN3 determined by the rate of cells filling the wound area is presented relative to that of 5637-control. Each value represents the mean (+SD) from at least three independent experiments. **P<0.01 (*vs.* control).

In order to confirm the specificity of the shRNA construct we used, the effects of a small interfering RNA (siRNA) for ZKSCAN3 on cell growth were assessed. We performed MTT assay in UMUC3 and 647V cells transfected with control-siRNA or ZKSCAN3-siRNA. Similar to the findings in ZKSCAN3-shRNA cells, ZKSCAN3-siRNA reduced the cell viability by 44-45%, compared with control-siRNA (Supplementary Figure S1). We further examined whether ZKSCAN3 overexpression could induce the growth of bladder cancer cells. As expected, overexpression of ZKSCAN3 in 5637 cells (Figure 5A) resulted in significant increases in their viability (Figure 5B) and migration (Figure 5C), compared with control cells.

### Anti-tumor activity of ZKSCAN3 silencing *in vivo*

**Figure 6:**
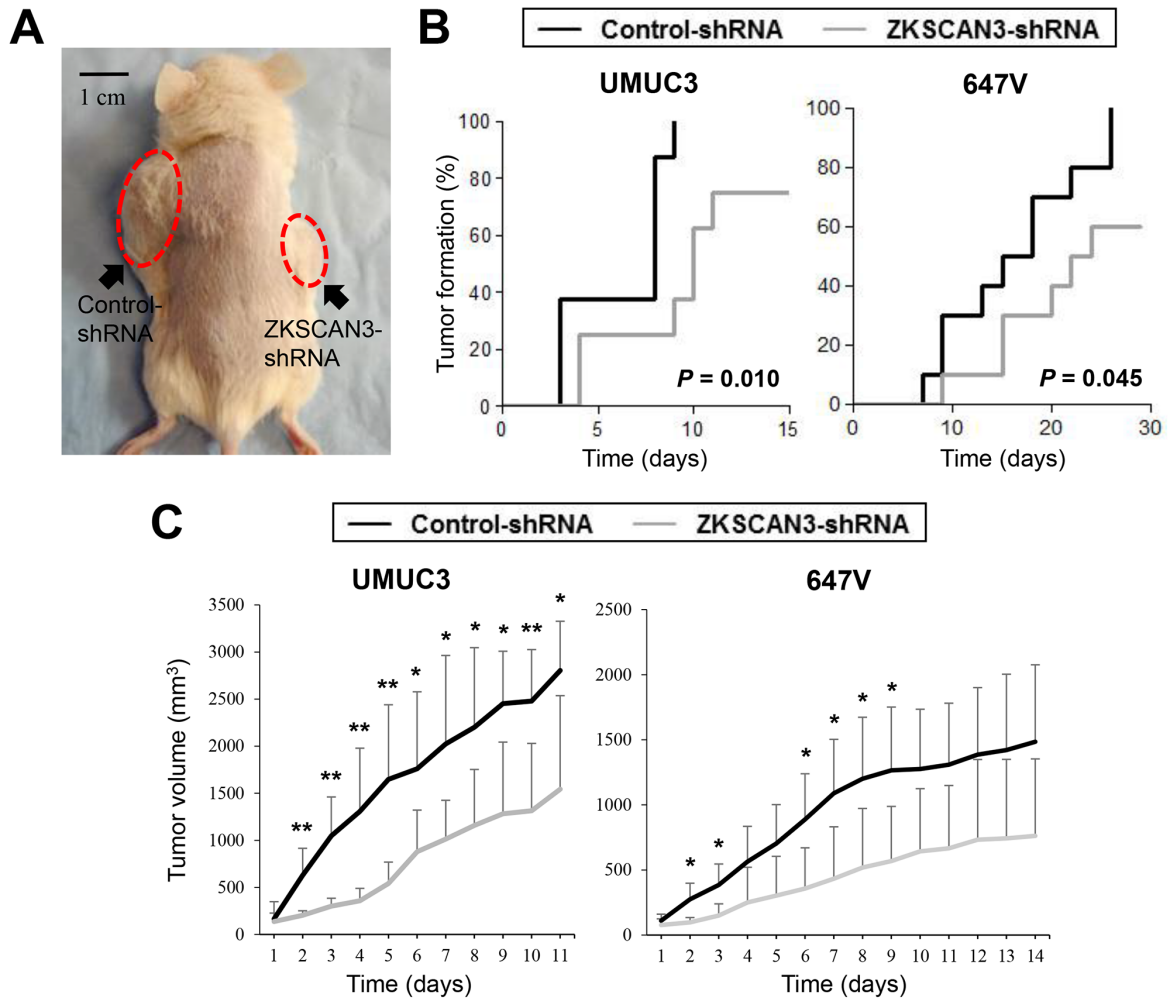
Effects of ZKSCAN3 inactivation on tumor growth in mouse xenograft models for bladder cancer **A.** UMUC3-control-shRNA/ZKSCAN3-shRNA and 647V-control-shRNA/ZKSCAN3-shRNA cells were implanted subcutaneously into the left/right flanks of NOD-SCID mice, respectively, and tumor formation and its growth were monitored. **B.** Kaplan-Meier curves and log-rank test according to the endpoint set as tumor volume exceeding 40 mm^3^. **C.** Tumor size (estimated volume of each tumor exceeded 40 mm^3^ at day 0) was subsequently monitored every day. Each value (n=4-6/group) represents the mean (+SD). *P<0.05 (control- *vs.* ZKSCAN3-shRNA). **P<0.01 (control- *vs.* ZKSCAN3-shRNA).

Next, we used mouse xenograft models to investigate the role of ZKSCAN3 in bladder tumor outgrowth *in vivo*. UMUC3-control-shRNA and UMUC3-ZKSCAN3-shRNA cells as well as 647V-ZKSCAN3-shRNA and 647V-control-shRNA cells were implanted subcutaneously into the flanks of immunocompromised mice (Figure 6A), and tumor development was monitored at the outset. ZKSCAN3 knockdown strikingly delayed the formation of UMUC3 or 647V xenograft tumors, compared with controls (Figure 6B). Following tumor formation (*i.e.* day 0 when the estimated tumor volume reached 40 mm^3^), its size was further monitored. As shown in Figure 6C, the inoculated ZKSCAN3-shRNA tumors were smaller than control-shRNA tumors [45% (UMUC3) and 49% (647V) decreases at the last days, respectively]. These *in vivo* data further suggest that ZKSCAN3 silencing inhibits the development and progression of bladder cancer.

### Effects of ZKSCAN3 on the expression of oncogenes and tumor suppressor genes

**Figure 7:**
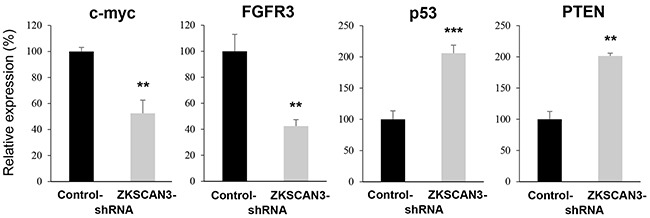
Effects of ZKSCAN3 inactivation on the expression of oncogenes and tumor suppressor genes Quantitative RT-PCR of *c-myc*, *FGFR3*, *p53*, and *PTEN* in UMUC3-control-shRNA and UMUC3-ZKSCAN3-shRNA cells. Expression of each specific gene was normalized to that of *GAPDH*. Transcription amount is presented relative to that of UMUC3-control line. Each value represents the mean (+SD) from at least three independent experiments. **P<0.01 (*vs.* control-shRNA). ***P<0.001 (*vs.* control-shRNA).

We finally compared the expression levels of oncogenes, such as c-myc and fibroblast growth factor receptor 3 (FGFR3), as well as tumor suppressor genes, such as p53 and phosphatase and tensin homolog (PTEN), all of which were known to involve urothelial tumorigenesis [2, 13, 14], in control-shRNA versus ZKSCAN3-shRNA cells. ZKSCAN3 silencing resulted in significant down-regulation of the expression of *c-myc* and *FGFR3* as well as significant up-regulation of the expression of *p53* and *PTEN* (Figure 7).

## DISCUSSION

To the best of our knowledge, the biological functions of zinc-finger transcription factors containing KRAB and SCAN domains in bladder cancer have not yet been characterized. In the present study, we have demonstrated that ZKSCAN3 expression is elevated in bladder cancer, compared with non-neoplastic urothelium, and that ZKSCAN3 silencing results in inhibition of bladder cancer cell proliferation, migration, and invasion. These findings in conjunction with our *in vivo* data suggest that ZKSCAN3 plays an important role in the development and progression of bladder cancer.

The expression of ZKSCAN3 mRNA and protein has been documented in three types of malignancies. First, using colorectal tissues, increased levels of *ZKSCAN3* gene expression were found in six of nine tumors, compared with corresponding benign controls [5]. Amplification of *ZKSCAN3* gene was also detected in some of the tumors with its overexpression [5]. Immunohistochemistry (IHC) in colorectal TMAs further showed higher ZKSCAN3 expression in invasive tumors than in non-invasive/intramucosal tumors [5]. Second, positive signals of ZKSCAN3 transcript and protein were detected in six of eight, including four with increased gene copy number, and eight of ten multiple myelomas, respectively, whereas normal plasma cells were negative for ZKSCAN3 [6]. Somatic mutation of *ZKSCAN3* gene was also found in multiple myeloma [15]. Third, ZKSCAN3 protein overexpression was observed in none of normal prostate tissues versus 49% of prostate cancer specimens, while *ZKSCAN3* gene amplification was detected in none of primary tumors, 20% of lymph node metastases, and 26% of bone metastases [7]. However, none of these studies have assessed the prognostic values of ZKSCAN3 expression or gene amplification in patients with respective malignancies [5–7]. Similar to the findings in these studies [5–7], we observed elevated expression of ZKSCAN3 mRNA and protein in bladder tumors, compared with control benign urothelial tissues. Nevertheless, ZKSCAN3 IHC in our bladder TMA showed positive staining in 84.4% of non-neoplastic urothelial tissues, possibly because these normal-appearing tissues used for IHC, as well as RT-PCR, were from the bladders from patients with bladder tumor and may not represent completely normal controls. In contrast, ZKSCAN3 was positive in only 2 (28.6%; 1 1+ and 1 2+) of 7 normal urothelial tissues included in the validation cohort of TMA. In addition, considerably elevated levels of ZKSCAN3 expression were observed in high-grade/muscle-invasive tumors, compared with low-grade/non-muscle-invasive tumors in the validation TMA, while there were no statistically significant associations between the status of ZKSCAN3 expression in these clinical samples we obtained at our institutions and tumor grade/stage. These results suggest the involvement of ZKSCAN3 in bladder tumorigenesis and tumor outgrowth. However, IHC in our TMA revealed no prognostic significance of ZKSCAN3 expression in these patients with urothelial tumor. Accordingly, functional dysregulation of ZKSCAN3, rather than the simple expression status, may be more closely related to bladder cancer progression. Meanwhile, copy number gain in the chromosome 6p22 region, where the *ZKSCAN3* gene resides, in bladder cancer has been shown to correlate with the risk of disease progression [11, 12].

In colon cancer cell lines, down-regulation of ZKSCAN3 via its shRNA or siRNA and its enforced expression inhibited and induced, respectively, colony formation as well as tumorigenicity and progression of xenografted tumors in mice [5]. ZKSCAN3-shRNA also suppressed the proliferation of myeloma cells and increased/decreased G1/S-G2 phase populations, respectively [6]. Additionally, using a prostate cancer line, ZKSCAN3 overexpression was shown to enhance cell detachment, cell migration, and tumorigenicity in xenograft-bearing mice and reduce apoptosis [7]. These findings, together with the profile of ZKSCAN3 expression and gene amplification in tissue specimens, suggested its role in tumor outgrowth. We further demonstrated, using bladder cancer lines, that ZKSCAN3 knockdown resulted in significant reduction of cell viability, colony formation, cell migration, cell invasion, and the expression of MMP-2/MMP-9, as well as significant induction of apoptosis. Correspondingly, ZKSCAN3 overexpression could strongly induce the proliferation and migration of bladder cancer cells. In mouse xenograft models for bladder cancer, ZKSCAN3 knockdown was also found to considerably retard tumor formation and subsequent tumor growth. Moreover, ZKSCAN3 knockdown was associated with down-regulated expression of oncogenes and up-regulated expression of tumor suppressor genes in bladder cancer cells. Thus, ZKSCAN3 was likely to promote bladder tumorigenesis and tumor progression.

There are no biomarkers widely used for detecting urothelial cancer and predicting patient outcomes. Meanwhile, no targeted agents have been approved for the treatment of bladder cancer. Previous studies have suggested that ZKSCAN3 directly or indirectly regulates the expression of various genes, such as CCND1, CCND2, EGFR, IGF-2, integrin-β4, NF-κB, and VEGF [6–8], all of which are known to involve tumorigenesis, tumor progression, and/or metastasis. In particular, EGFR and VEGF have been validated as promising molecular targets for the inhibition of bladder cancer growth in preclinical studies [16]. In addition, ZKSCAN3 has recently been implicated in the regulation of autophagy [9, 10] whose defects are also linked to cancer. We found that ZKSCAN3 silencing correlated with the down-regulation of NF-κB expression in bladder cancer cells. Based on the previous and current findings, ZKSCAN3 may represent not only a useful biomarker of bladder cancer but also a promising therapeutic target.

In summary, ZKSCAN3 appears to be activated in bladder cancer and promotes tumor growth. Our findings may therefore offer a potential therapeutic strategy for bladder cancer via targeting ZKSCAN3 signaling. Further assessment of the functions of ZKSCAN3 is required to determine its biological significance in bladder cancer. Mechanistic details underlying the promotion of tumor development and progression by ZKSCAN3 also need to be further characterized.

## MATERIALS AND METHODS

### Cell culture

UMUC3, 5637, and SVHUC cell lines were originally obtained from the American Type Culture Collection. 647V cell line was used in our previous studies [17–21]. All these lines were recently authenticated, using GenePrint 10 System (Promega), by the institutional core facility. SVHUC cells and other cell lines were cultured in F-12K (Mediatech) and Dulbecco's modified Eagle's medium (Mediatech), respectively, all supplemented with 5% fetal bovine serum (FBS), penicillin (100 units/mL), and streptomycin (100 units/mL) at 37°C in a humidified atmosphere of 5% CO_2_.

### Stable cell lines

A shRNA plasmid targeting human ZKSCAN3 (sc-95093-SH; Santa Cruz Biotechnology) or a non-silencing control shRNA plasmid (sc-108060; Santa Cruz Biotechnology) was transfected into UMUC3 and 647V, using Lipofectamine^®^ 2000 transfection reagent (Life Technologies). Selection of stable clones was carried out with puromycin (Sigma) treatment at a concentration of 6 μg/mL, as described previously [20]. In addition, a ZKSCAN3-siRNA (#4392420; Ambion) and a ZKSCAN3 construct (RC202971; OriGene) were transiently transfected into UMUC3/647V and 5637 cells, respectively.

### Bladder TMA

We retrieved bladder tissue specimens obtained by transurethral resection performed at the Johns Hopkins Hospital or University of Rochester Medical Center. All the sections were reviewed for confirmation of original diagnoses, according to the 2004 World Health Organization/International Society of Urological Pathology classification system for urothelial neoplasms. Appropriate approval from the institutional review board at each institution was obtained before construction and use of the TMAs. Bladder TMAs, consisting of 148 cases of urothelial neoplasm, were constructed from formalin fixed paraffin embedded specimens, as described previously [22]. These patients included 113 men and 35 women with a mean/median age of 66.0/69 years (range: 26-89). All 64 patients with muscle-invasive tumor ultimately underwent cystectomy. None of the patients had received therapy with radiation or anti-cancer drugs prior to the collection of the tissues included in the TMAs. For the validation study of ZKSCAN3 protein expression in tissue specimens, we also used the bladder cancer high density tissue array (BL2081, US Biomax).

### Bladder cancer tissues for PCR analysis

We collected a total of 51 cases of tissue specimens (bladder tumor histologically diagnosed as urothelial carcinoma and adjacent normal-appearing bladder) obtained by transurethral resection/cold-cup biopsy performed at Yokohama City University Medical Center, immediately frozen in liquid nitrogen, and stored at -80°C. Appropriate approval for the use of these specimens was obtained from the institutional review board at our institution. The patients included 39 men and 12 women with a mean/median age of 70.7/72 years (range: 38-100). None of these patients had received any preoperative anticancer therapies.

### Western blotting

Protein extraction and western blotting were performed, as described previously [18–21] with minor modifications. Equal amounts of protein (30-50 μg) obtained from cell extracts were separated in 10% sodium dodecyl sulfate (SDS)-polyacrylamide gel electrophoresis (PAGE) and transferred to polyvinylidene difluoride membrane (Immun-Blot PVDF Membrane, Bio-Rad) by electroblotting. Specific antibody binding was detected, using an anti-ZKSCAN3 antibody (TA308508; dilution 1:500; OriGene) or an anti-GAPDH antibody (clone 6C5; dilution 1:5000; Santa Cruz Biotechnology), and a secondary antibody (rabbit IRDye^®^ 800CW, LI-COR), followed by scanning with an infrared imaging system (Odyssey, LI-COR).

### RT and real-time PCR

Total RNA (0.5 μg) isolated from cultured cells using TRIzol (Invitrogen) or tissue specimens using ISOGEN (Nippon Gene) was reverse transcribed using 1 μM oligo (dT) primers and 4 units of Omniscript reverse transcriptase (Qiagen) in a total volume of 20 μL. Real-time PCR was then performed, using iQ™ SYBR^®^ Green Supermix (Bio-Rad) or Fast SYBR^®^ Green Master Mix (Thermo Fisher Scientific). The following primer pairs were used for RT-PCR: *ZKSCAN3* (forward, 5′-CCCAGGGTCACAAAGTAGCC-3′; reverse, 5′-GGA CTCTGGAGTAAGCCTAGAA-3′; or forward, 5′-TGA CAGCTACTAGGCTCACAT-3′; reverse, 5′-GCAA GTCCCTAACCTTAGTCTGC-3′), *NF-κB* (forward, 5′-AACAGAGAGGATTTCGTTTCCG-3′; reverse, 5′-TTTGACCTGAGGGTAAGACTTCT-3′); *MMP-2* (forward, 5′-TACAGGATCATTGGCTACACACC-3′; reverse, 5′-GGTCACATCGCTCCAGACT-3′); *MMP-9* (forward, 5′-TGTACCGCTATGGTTACACTCG-3′; reverse, 5′-GGCAGGGACAGTTGCTTCT-3′); *c-myc* (forward, 5′-ACCAGATCCCGGAGTTGGAA-3′; reverse, 5′-CGTCGTTTCCGCAACAAGTC-3′); *FGFR3* (forward, 5′-TGCGTCGTGGAGAACAAGTTT-3′; reverse, 5′-GCACGGTAACGTAGGGTGTG-3′); *p53* (forward, 5′-TCTGGGACAGCCAAGTCTGT-3′; reverse, 5′-GGAGTCTTCCAGTGTCATGA-3′); and *PTEN* (forward, 5′-GTTTACCGGCAGCATCAAAT-3′; reverse, 5′-CCCCCACTTTAGTGCACAGT-3′). *GAPDH* (forward, 5′-CTCCTCCACCTTTGACGCTG-3′; reverse, 5′-CATACCAGGAAATGAGCTTGACAA-3′) was used as an internal control.

### IHC

IHC was performed on the sections (5 μm thick) from the bladder TMAs, using a primary antibody to ZKSCAN3 (TA308508; dilution 1:50), as described previously [19, 20]. All stains were manually quantified by a single pathologist (H.M.) blinded to sample identify. The German immunoreactive scores (range: 0-12) calculated by multiplying the percentage of immunoreactive cells (0%=0; 1-10%=1; 11-50%=2; 51-80%=3; 81-100%=4) by staining intensity (negative=0; weak=1; moderate=2; strong=3) were considered negative (0; 0-1), weakly positive (1+; 2-4), moderately positive (2+; 6-8), and strongly positive (3+; 9-12).

### Reporter gene assay

Cells at a density of 50-70% confluence in 24-well plates were co-transfected with 250 ng of NF-κB-Luc reporter plasmid DNA (Signosis) and 2.5 ng of pRL-TK plasmid DNA, using GeneJuice (Novagen). After 24 hours of transfection, the cells were harvested, lysed, and assayed for luciferase activity determined using a Dual-Glo^®^ Luciferase Assay System (Promega) and luminometer (FLUOstar Omega, BMG Labtech).

### Cell proliferation

We used MTT assay to assess cell viability. Cells (0.5-1 × 10^3^) seeded in 96-well tissue culture plates were incubated for 24-144 hours, and at the end of the culture 10 μL MTT stock solution (5 mg/mL; Sigma) was added to each well with 100 μL of medium for 4 hours at 37°C. The medium was replaced with 100 μL dimethyl sulfoxide, followed by incubation for 5 minutes at room temperature. The absorbance was then measured at a wavelength of 570 nm with background subtraction at 655 nm using luminometer (FLUOstar Omega).

### Colony formation

Cells (5 × 10^2^) seeded in 12-well plates were allowed to grow until colonies in the control well were easily distinguishable. The cells were then fixed with methanol and stained with 0.1% crystal violet. The number of colonies and their areas were quantitated using ImageJ software (National Institutes of Health).

### Apoptosis and cell cycle analysis

The TUNEL assay was performed on cell-burdening coverslips, using the DeadEnd Fluorometric TUNEL system (Promega), followed by counterstaining for DNA with 4′,6′-diamidino-2-phenylindole (DAPI). Apoptotic index was determined in the cells visualized by the fluorescence microscopy (Invitrogen EVOS FL Auto Cell Imaging System). For cell cycle analysis, flow cytometry was performed in cells (1 x 10^6^/10-cm dish) cultured for 24 hours, harvested with trypsin, fixed in 70% ethanol, and stained with propidium iodide (PI) buffer (50 μg/mL) for 30 minutes. Cellular PI content was measured on a Guava PCA-96 Base System^TM^ flow cytometer (EMD Millipore) equipped with a green laser at 532 nm wavelength. Data were analyzed, using the Guava Cell Cycle software (EMD Millipore).

### Cell migration

A scratch wound healing assay was adapted to evaluate the ability of cell migration. Cells at a density of 80-90% confluence in 6-well plates were scratched manually with a sterile 200 μL plastic pipette tip, cultured for 24 hours, fixed with methanol, and stained with 0.1% crystal violet. The width of the wound area was quantitated, using the ImageJ.

### Cell invasion

Cell invasiveness was determined, using a Matrigel (60 μg; BD Biosciences)-coated transwell chamber (8.0 μm pore size polycarbonate filter with 6.5 mm diameter; Corning), as described previously [19, 20]. Briefly, cells (5 × 10^4^) in 100 μL of serum-free medium were added to the upper chamber of the transwell, whereas 600 μL of medium containing 5% FBS was added to the lower chamber. After incubation for 24 hours at 37°C in a CO_2_ incubator, invaded cells were fixed, stained with 0.1% crystal violet, and counted under a light microscope.

### Mouse xenograft model

Animal protocols in accordance with the National Institutes of Health Guidelines for the Care and Use of Experimental Animals were approved at our institution. Cells (1 × 10^6^/100 μL/site) resuspended in Matrigel (BD Biosciences) were subcutaneously injected into the flank of 6-week-old male immunocompromised NOD-SCID mice, as described previously [19, 20, 23]. Serial caliper measurements of perpendicular diameters were used to calculate tumor volume by the following formula: (short diameter)^2^ x (longest diameter) × 0.5.

### Statistical analysis

The Fisher exact test or the χ^2^ test was used to evaluate the associations between categorized variables. The numerical data were compared by Student's *t*-test or Mann-Whitney *U* test. Survival rates in patients were calculated by the Kaplan-Meier method and comparison was made by log-rank test. *P* values less than 0.05 were considered to be statistically significant.

## SUPPLEMENTARY MATERIALS FIGURE AND TABLE


