# Supplementary material for: ZKSCAN3 promotes bladder cancer cell proliferation, migration, and invasion
Source: Oncotarget. 2016 Jul 18;7(33):53599–610. doi: 10.18632/oncotarget.10679 (PMC5288208; doi:10.18632/oncotarget.10679)
Supplement: Supplementary file 1 [file oncotarget-07-53599-s001.pdf]

ZKSCAN3 promotes bladder cancer cell proliferation, migration, and invasion

SUPPLEMENTARY FIGURE AND TABLE

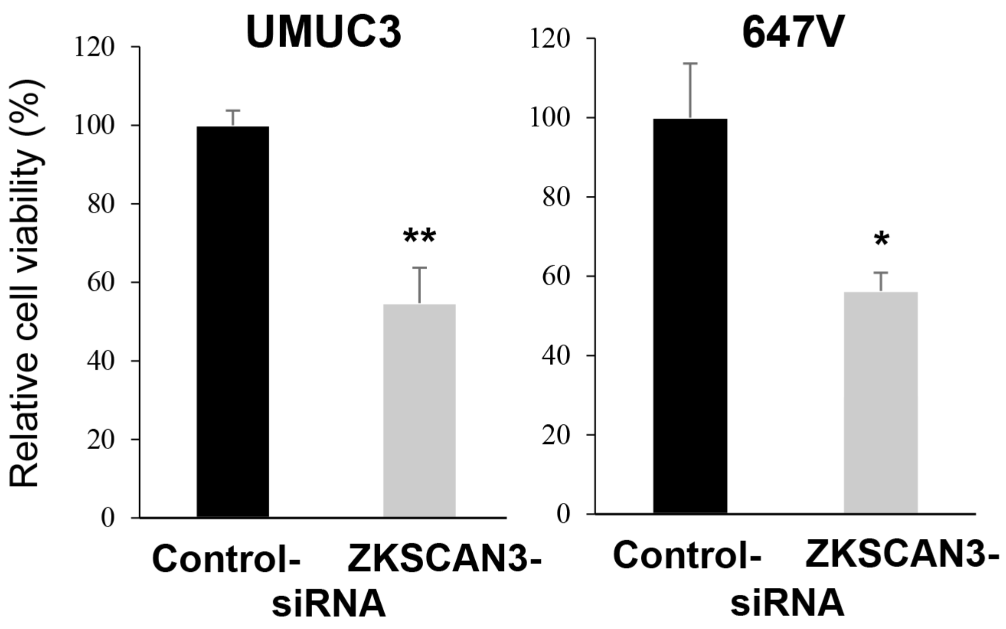

Supplementary Figure S1: Effects of ZKSCAN3 inactivation on bladder cancer cell proliferation. MTT assay in UMUC3-control-siRNA/ZKSCAN3-siRNA and 647V-control-siRNA/ZKSCAN3-siRNA cultured for 4 days. Cell viability is presented relative to that of each control line. Each value represents the mean (+SD) from at least three independent experiments. *$P<0.05$ (*vs.* control-siRNA). **$P<0.01$ (*vs.* control-siRNA).

Supplementary Table S1: Correlation of ZKSCAN3 expression in bladder tumors on a validation set of tissue microarray

	n	Expression levels				*P*-value	
		Negative	Positive				
		0	1+	2+	3+	0 *vs* 1+/2+/3+	0/1+/2+ *vs* 3+
**Tissue**							
Normal urothelium	7	5 (71.4%)	1 (14.3%)	1 (14.3%)	0 (0%)	<0.001	0.097
Urothelial neoplasm	159	5 (3.1%)	32 (12.6%)	65 (40.9%)	57 (35.8%)		
**Gender**							
Male	124	4 (3.2%)	27 (21.8%)	48 (38.7%)	45 (36.3%)	1.000	0.845
Female	35	1 (2.9%)	5 (14.3%)	17 (48.6%)	12 (34.3%)		
**Tumor grade**[a]							
LG	61	3 (4.9%)	17 (27.9%)	29 (47.5%)	12 (19.7%)	0.373	0.001
HG	98	2 (2.0%)	15 (15.3%)	36 (36.7%)	45 (45.9%)		
**Tumor invasiveness**							
NMI	67	3 (4.5%)	12 (17.9%)	34 (50.7%)	18 (26.9%)	0.651	0.047
MI	92	2 (2.2%)	20 (21.7%)	31 (33.7%)	39 (42.4%)		

LG: Low-grade urothelial carcinoma; HG: High-grade urothelial carcinoma; NMI: Non-muscle-invasive tumor; MI: Muscle-invasive tumor.

[a]All the tumors were re-graded according to the 2004 World Health Organization/International Society of Urological Pathology classification system by a pathologist (H.M.).
